# *Elthusa nierstraszi* nom. n., the replacement name for *Elthusa parva* (Nierstrasz, 1915), a junior secondary homonym of *Elthusa parva* (Richardson, 1910) (Isopoda, Cymothoidae)

**DOI:** 10.3897/zookeys.619.10143

**Published:** 2016-09-27

**Authors:** Kerry A. Hadfield, Niel L. Bruce, Nico J. Smit

**Affiliations:** 1 Water Research Group (Ecology), Unit for Environmental Sciences and Management, Potchefstroom Campus, North-West University, Private Bag X6001, Potchefstroom, 2520, South Africa; 2 Museum of Tropical Queensland, 70–102 Flinders Street, Townsville, Australia 4810

**Keywords:** Meinertia parva, Livoneca parva, ﻿junior homonym, new combination, marine fish parasite

## Abstract

The recent transfer of *Elthusa parva* (Richardson, 1910) from *Ceratothoa* created a homonymy with *Elthusa parva* (Nierstrasz, 1915). *Elthusa parva* (Richardson, 1910) has priority and *Elthusa nierstraszi* **nom. n.** is proposed as the new replacement name for the junior secondary homonym *Elthusa parva* (Nierstrasz, 1915).

## Introduction

Hadfield et al. (2016) in redescribing poorly characterised species of *Ceratothoa* Dana, 1852 transferred the species *Ceratothoa parva* (Richardson, 1910) to *Elthusa* Schioedte & Meinert, 1884. In so doing *Elthusa parva* (Nierstrasz, 1915) was rendered a junior secondary homonym.

According to Articles 23.3.5, 52 and 60.3 of the International Code of Zoological Nomenclature (Anon 1999) *Elthusa parva* (Richardson, 1910) (originally in combination with *Meinertia* Stebbing, 1893) has priority and a new name *Elthusa nierstraszi* **nom. n.** is proposed for *Elthusa parva* (Nierstrasz, 1915) to remove the homonymy.

**Abbreviations.** RMNH – Rijksmuseum voor Natuurlijke Historie (Naturalis Biodiversity Center).

## Taxonomic history

***Meinertia parva* Richardson, 1910**: 21, fig. 20.

*Codonophilus parvus*: Nierstrasz 1931: 132.

*Ceratothoa parva*: Trilles 1994: 127.

*Elthusa parva*: Hadfield et al. 2016: 75, fig. 12.

***Livoneca parva* Nierstrasz, 1915**: 98, pl. IV, figs. 18, 19; 1931: 143.

*Elthusa parva*: Bruce 1990: 287.

*Lironeca parva*: Trilles 1994: 182.

**Suborder Cymothoida Wägele, 1989**

**Superfamily Cymothooidea Leach, 1814**

**Family Cymothoidae Leach, 1814**

**Genus *Elthusa* Schioedte & Meinert, 1884**

### Elthusa nierstraszi nom. n.

Taxon classificationAnimaliaIsopodaCymothoidae

Livoneca parva Nierstrasz, 1915: 98–99, pl. IV, figs. 18, 19; 1931: 143.
Elthusa parva : Bruce 1990: 287; Sidabalok 2013: 56.
Lironeca parva : Trilles 1994: 182.

#### Holotype.

Single ovigerous female (11 mm long; 5 mm wide), Kisser, Indonesia, coll. K. Schädler, 1898 (RMNH.CRUS.I.71).

#### Distribution.

Kisser (= Kisar?) Island, Moluccas, Indonesia (Nierstrasz 1915).

#### Hosts.

Unknown.

#### Etymology.

The new name honours the original author Hugo Frederik Nierstrasz (1872–1937).

#### Remarks.

*Elthusa nierstraszi* nom. n. replaces the name *Elthusa parva* (Nierstrasz, 1915), originally *Livoneca parva* Nierstrasz, 1915. *Elthusa nierstraszi* nom. n. has mesially directed, narrow anterolateral margins on pereonite 1; pereonite 7 not overlapping pleonite 1; pleonites all subequal in width and narrower than the pereon; and the posterior margin of the pleotelson forms a caudomedial point.

The cover date for Trilles’ (1994) ‘*Prodromus*’ was 1991, but the work was most likely completed prior to that of Bruce (1990) and Trilles was simply retaining *Livoneca parva* in its original combination (albeit using the alternative incorrect spelling *Lironeca*; see Anon 1996). Prior to Bruce (1990) the name *Livoneca* (also as *Lironeca*) was largely used in the sense of *Elthusa*, and there is no possibility that Nierstrasz’s species would remain in or be returned to *Livoneca*, a strictly New World genus.

## Supplementary Material

XML Treatment for Elthusa nierstraszi
